# Effects of Vehicle Load on Emissions of Heavy-Duty Diesel Trucks: A Study Based on Real-World Data

**DOI:** 10.3390/ijerph18083877

**Published:** 2021-04-07

**Authors:** Xin Wang, Guohua Song, Zhiqiang Zhai, Yizheng Wu, Hang Yin, Lei Yu

**Affiliations:** 1 Key Laboratory of Transport Industry of Big Data Application Technologies for Comprehensive Transport, Beijing Jiaotong University, 3 Shangyuancun, Haidian District, Beijing 100044, China; 18114044@bjtu.edu.cn (X.W.); zhaizq@bjtu.edu.cn (Z.Z.); wuyizheng@bjtu.edu.cn (Y.W.); Lei.Yu@tsu.edu (L.Y.); 2 Department of Civil & Mineral Engineering, University of Toronto, 35 St. George Street, Toronto, ON M5S 1A4, Canada; 3 State Environmental Protection Key Laboratory of Vehicle Emission Control and Simulation (VECS), Chinese Research Academy of Environmental Sciences, 8 Dayang Fang, Chaoyang District, Beijing 100012, China; yinhang@vecc-mep.org.cn; 4 Department of Transportation Studies, Texas Southern University, 3100 Cleburne Avenue, Houston, TX 77004, USA; 5 School of Traffic and Transportation, Xuchang University, Xuchang 461000, China

**Keywords:** diesel semi-trailer towing trucks, vehicle load, STP distribution, emission rates, emission characteristics

## Abstract

Vehicle loads have significant impacts on the emissions of heavy-duty trucks, even in the same traffic conditions. Few studies exist covering the differences in emissions of diesel semi-trailer towing trucks (DSTTTs) with different loads, although these vehicles have a wide load range. In this context, the operating modes and emission rates of DSTTTs were analyzed under varying loads scenarios to understand the effect of vehicle loads on emission factors. First, second-by-second field speed data and emission data of DSTTTs with different loads were collected. Then, the methods for calculating the scaled tractive power (STP) and the emissions model for DSTTTs were proposed to evaluate the effect of different loading scenarios. The STP distributions, emission rate distributions, and emission factor characteristics of different loaded trucks were analyzed and compared. The results indicated that the STP distributions of DSTTTs that under the unloaded state were more narrow than those under fully loaded or overloaded conditions. The emission rates of carbon dioxide (CO_2_), carbon monoxide (CO) and total hydrocarbon (THC) for DSTTTs under a fully loaded state were significantly higher than those under an unloaded state. However, due to the influence of exhaust temperature, the emission rates of nitrogen oxides (NO_x_) among fully loaded trucks were lower than those under the unloaded state when STP bin was above 4 kW/ton. The emission factors of CO_2_, CO, THC, and NO_x_ for fully loaded trucks demonstrated the largest increases at low-speed intervals (0–30 km/h), which rose by 96.2%, 47.9%, 27.8%, and 65.2%, respectively.

## 1. Introduction

Heavy-duty trucks (HDTs) are major contributors to urban traffic pollution. In China, HDTs emit 16.8% of carbon monoxide (CO), 6.9% of total hydrocarbon (THC), 57.8% of nitrogen oxides (NO_x_), and 66.3% of particulate matter (PM) of the total motor vehicle emissions, despite only constituting 3.1% of the vehicle population [1]. Hence, to control urban traffic emissions, it is crucial to accurately assess and strictly monitor the emissions of heavy-duty diesel trucks (HDDTs). However, HDDTs have a wide load range—for example, diesel semi-trailer towing trucks (DSTTTs) have a load difference of 34 tons between unloaded and fully loaded conditions—and the variety in load states can significantly impact the accuracy of emissions estimates [2,3,4]. Few existing emission models are available to calculate the emissions of DSTTTs while considering different load states and, as a result, emissions are often misestimated, which may mislead or complicate emission control policies. HDDTs contribute the most NO_x_ in urban environments and it is predicted that their population and vehicle kilometers traveled will continue to rise, leading to ever-increasing emissions [5,6]. Thus, the accurate estimation of HDDTs emissions is crucial for the proper design of management and control schemes for HDDTs. This study sought to analyze the effect of load on emission assessments and attempted to widen the applicability of the HDDTs emission model.

HDDTs emissions measurements are typically performed using engine dynamometers, tunnel studies, remote sensing, and portable emissions monitoring systems (PEMS) [7,8,9]. The engine dynamometer test cycle is based on a specific driving cycle in the laboratory, and it is typically considered that such cycles do not represent the full range of real-world vehicle operating modes [10]. Meanwhile, tunnel testing is performed by installing devices within the tunnel to test the average emissions of all passing vehicles. In this regard, they may not be representative of emissions under all field operating conditions, and make it difficult to distinguish vehicle types [11]. Remote sensing measurements can offer a snapshot of active vehicle emission concentrations at a specific location and, therefore, might not represent an entire operating cycle [12]. As compared with the above emissions measurement methods, PEMS are able to obtain emissions data from vehicles operating in real-time on the field road network, taking into account the impact of changing traffic conditions.

Some researchers have studied emission characteristics based on PEMS to assess vehicle emissions better [13,14]. Still, as different vehicle loads have a critical role to play on the emissions of HDDTs [15,16], it is vital to explore the operating modes and emission characteristics of these trucks under varying loads. Yao et al. [17] tested the emissions of on-road HDDTs (with a gross vehicle weight of 16.0 tons) under 0%, 50%, and 100% loads using PEMS. The results showed that emission factors of NO_x_ and PM for the trucks when half-loaded were 43% and 59% higher than those obtained when the trucks were unloaded, and 62% and 44% higher when fully loaded. Elsewhere, Frey et al. [3] collected emissions data for diesel-fueled tandem trucks (with a gross vehicle weight of 29.0 tons) under different loads using PEMS and studied the emission variations between unloaded and fully loaded trucks. Their results indicated that the difference for loaded trucks versus unloaded trucks is 44% for CO_2_, 78% for NO_x_, 23% for PM, 30% for HC and 22% for CO. Song et al. [18] obtained the emissions data of two HDDTs (with a gross vehicle weight of 25.0 tons) with different loads (empty, half, and full loads). This study found that the NO_x_, CO, and THC emission factors of the tested vehicles when half loaded and fully loaded were 18% to 41%, 6% to 67%, and 37% to 125% higher than those obtained when the trucks were not loaded, respectively. During the field testing, it is challenging to maintain the same transport scenario for vehicles for different loads based on the PEMS test alone. Therefore, it is impossible to perform a comparable and consistent analysis for HDDTs emissions at various load states.

The vehicle specific power (VSP) distributions could be used to further characterize traffic conditions and vehicle emissions. The VSP or STP was designed to reflect the engine power required for the vehicle to overcome aerodynamic, rolling resistance, and rotational forces, so as to move the vehicle forward on the actual road [19]. Song et al. [20] characterized the VSP distribution of light-duty vehicles on urban restricted-access roadways and established a relationship between the VSP distribution and the average operating speed. Lai et al. [21] constructed a city-specific driving cycle based on the STP distribution for transit buses and then estimated vehicle emissions. Li et al. [22] established VSP binning division using field data for urban transit buses to better reflect these vehicles’ operating characteristics. Zhang et al. [4] proposed a method for calculating the VSP values and developed an emissions model for heavy-duty refuse trucks (with a gross vehicle weight of 15.5 tons) to analyze the impact of empty and full loads on emissions.

It is only one-sided to consider the effect of changes in VSP distribution on the emissions of various loaded DSTTTs [23]. For DSTTTs equipped with a selective catalytic reduction (SCR) system, the exhaust temperature seriously affects the NO_x_ emission rate [24]. The activity level the catalyst rises and then falls with activity level changes in exhaust temperature, resulting in the variations in the NO_x_ conversion efficiency [25,26]. The heavier vehicle load causes the NOx concentration at the inlet of the SCR device to increase, and at the same time, it also leads the exhaust temperature to rise. Under the simultaneous effects of these two factors, the ultimate NO_x_ emission rate changes caused by various load conditions needs to be further analyzed.

Existing studies suggest that vehicle loads have a significant effect on the emissions of HDDTs. Most studies on HDDTs can be mainly divided into two categories, one is to analyze the emission characteristics directly based on the emission data, the other is to study the difference of VSP distribution based on the vehicle operation data. The studies based on emission data mainly uses the data collected by the engine dynamometer or PEMS. The former can hardly reflect the effect of truckloads on emissions in the real-world, and the latter approach does not ensure the accurate analysis of emissions for different load levels for the same traffic conditions. Meanwhile, a few VSP-based studies on HDDT emissions mainly concentrate on the influence of VSP distributions on emissions for different loaded trucks. It does not take into account the fluctuation of exhaust temperature, which affects the differences in emission rates corresponding to the VSP distributions. Besides, few studies have assessed the emission differences of DSTTTs with a wide load range.

In this context, the method for calculating the scaled tractive power (STP) and the emissions model for DSTTTs was developed based on second-by-second field speed data collected using on-board diagnostics (OBD) devices and emission data obtained by using PEMS instruments. Then, the operating modes and emission rates for DSTTTs were analyzed under various loads further to understand the effect of truck loads on emission factors. Finally, emission factor differences of CO_2_, CO, THC, and NO_x_ under different load conditions were compared.

## 2. Materials and Methods

**Figure 1:**
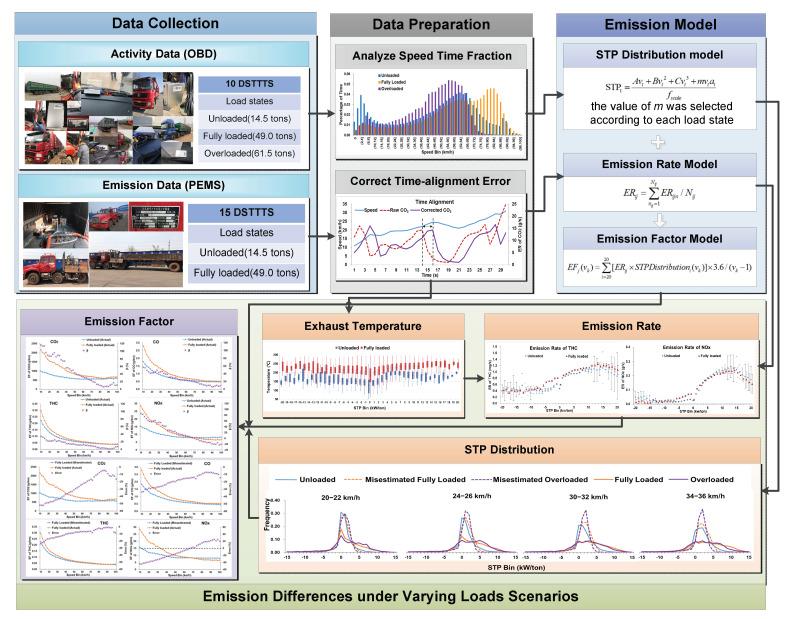
Framework of this study.

The methods adopted for this study consisted of five sections, as shown in Figure 1. First, emission data and the field operating data were acquired from 25 DSTTTs under different load conditions. The vehicle weights were approximately 14.5 tons for unloaded trucks, 49.0 tons for fully loaded trucks, and 61.5 tons for overloaded trucks. Second, the data processing methods of STP distribution and the emission rate model were designed. Third, the STP distribution in each speed bin of DSTTTs under different load states was compared and explained. Fourth, emission rate differences in CO_2_, CO, THC, and NO_x_ under various load states were analyzed. Finally, the emission factors of CO_2_, CO, THC, and NO_x_ under various load states were modeled as a function of speed.

### 2.1. Data Preparation

#### 2.1.1. Data Collection and Tested Vehicles

Due to the limitation of data acquisition conditions, vehicle data were obtained in two ways. One method was the acquisition of second-by-second activity data of the DSTTTs, which was used to study the operation difference between various load states under the same traffic modes; the other method was by gathering vehicle emissions data, which was used to establish an emission rate model to analyze the differences in emission rates among trucks with varying loads.

A total of 10 DSTTTs were identified in Shandong Province to gather second-by-second operating speed data using OBD devices to study STP distribution characteristics for DSTTTs under different loads. The speed data collected for this study covered three conditions: unloaded, fully loaded, and overloaded. The payloads information of DSTTTs was obtained from a weighbridge prior to each test. The test vehicle information was listed in Table 1. The gathered activity data included 1,692,632 records of second-by-second DSTTTs trajectories under the unloaded condition, 1,454,043 records under the fully loaded condition, and 452,502 records under the overloaded condition. Study data were collected from 28 March 2020, to 10 June 2020.

Meanwhile, truck emissions data were collected using a PEMS instrument (HORIBA OBS-ONE, Japan) from 23 August 2018 to 10 September 2018, in Heibei province. The emissions data collected covered the unloaded condition and fully loaded condition. The detailed information on DSTTTs used for emissions testing is provided in Table 2. The DSTTTs weights were roughly 14.5 tons for unloaded trucks and 49.0 tons for fully loaded vehicles. The quality control of the collected data was carried out through speed and acceleration. One was to eliminate data with a speed exceeding 120 km/h, and the other was to filter the data by using the 98% acceleration distribution quantile as the threshold. After rigorous data quality control procedures, a total of 153,215 valid second-by-second emissions data were retained, including 101,432 records for empty trucks and 51,783 records for fully loaded trucks.

#### 2.1.2. Data Preparation

**Figure 2:**
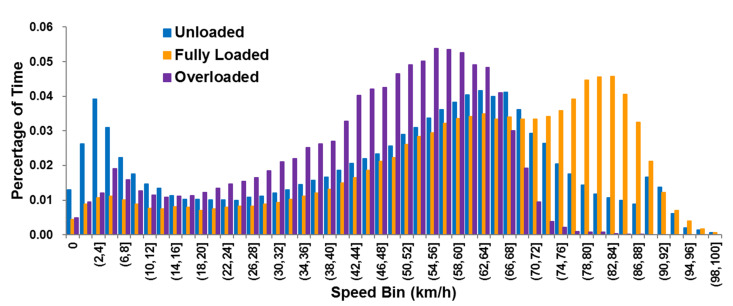
Time percentage of operating speed for trucks under unloaded, fully loaded and overloaded states in each speed bin.

After controlling for data quality, the interval of 2 km/h was used to divide the speed bin and the time proportion of samples in each speed interval analyzed. As shown in Figure 2, the average speeds of unloaded, fully loaded, and overloaded trucks were 48.68 km/h, 60.45 km/h, and 45.39 km/h, respectively. The speed distribution of unloaded trucks was more scattered and the time proportion of samples in each speed bin below 92 km/h exceeded 1%. There were three peaks in the speed distribution for the unloaded trucks, which were 2 to 4 km/h, 62 to 64 km/h, and 88 to 90 km/h, respectively. This speed distribution could be explained by the idea that trucks queuing up to load in a logistics park led to long-term low-speed operation, while unloaded vehicles typically use unrestricted access roads to get to closer loading points, resulting in vehicles in medium speed operation, or using restricted access roads to return from further destinations after unloading to make them operating at a relatively high speed. Fully loaded trucks operated mainly in the speed ranges of 58 to 88 km/h. This situation is likely because the trucks reached their destination via the restricted access roads after loading cargo. Finally, the speed data of overloaded trucks were more concentrated than those with the no-load and a full load. The overloaded vehicles mainly operated in the speed range of 42 to 66 km/h and there was relatively limited operation data available in the high-speed range. These trucks typically drive on unrestricted-access roads to avoid administrative penalties and, due to vehicle performance limitations and driver safety considerations, the operating speed of such overloaded trucks was relatively low.

**Figure 3:**
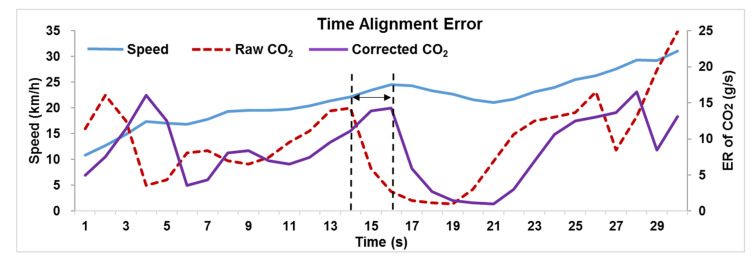
Time alignment error of CO_2_ emission data steam.

A time-alignment error typically existed in data streams from the exhaust gas collection system and the global positioning system or OBD devices’ speed recorder. In this study, different data sources were used to calculate STP and emission rates and there was a time lag reflecting different operating modes of the vehicle at the same time. For example, in Figure 3, the CO_2_ emission data of trucks lagged the operating speed at the same time. This study presumed that the time of the STP was considered as the basic timeline in the calibration method. Then, the time alignment sequence was readjusted based on the maximum correlation coefficient between the STP and emission data. It should be emphasized that the various pollutants must be modified individually with operating speed due to discrepancies in time-alignment errors for different pollutants. The consistency of CO_2_, CO, THC, NO_x_ and STP values were significantly improved following correction for time-alignment error.

### 2.2. Calculation of STP Distribution and Emission Rate

To avoid the impact of road slope on the analysis of truck activity loads, sloped roads, both uphill and downhill with slopes exceeding 1.5%, were marked during the data quality control process. These tagged data were then excluded when performing data analysis to calculate STP values. The formula was calculated using Equation (1) as follows without the parameter of road gradient:

(1) $${\mathrm{STP}}_{t}=\frac{Av_{t}+Bv_{t}{}^{2}+Cv_{t}{}^{3}+mv_{t}a_{t}}{f_{scale}}$$

where STP_t_ = STP value at time *t* (kW/ton), *v_t_* = operating speed at time *t* (m/s), *a_t_* = transient acceleration of truck operation at time *t* (m/s^2^), *m* = gross truck weight (tons), *A* = rolling resistance coefficient (kW-s/m), *B* = rotational resistance coefficient (kW-s^2^/m^2^), *C* = aerodynamic drag coefficient (kW-s^3^/m^3^), and *f_scale_* = scaling factor, with a fixed value of 17.1 being suggested [27].

As shown in Equation (1), the A, B, and C were constant for a specific vehicle type. Moreover, the speed, acceleration, and gross vehicle weight determined its calculated STP values. Therefore, for the same operation mode, an increase of m led to a proportional increase in STP values. In this study, the values of m used for calculating STP values for DSTTTs that were unloaded, fully loaded, and overloaded were 14.5 tons, 49.0 tons, and 61.5 tons, respectively. The method for obtaining the STP distribution and emission rates under different loads was as follows:

(1) Calculating second-by-second STP values of trucks under unloaded, fully loaded, and overloaded conditions using Equation (1) where the value of m was selected according to each load state.
(2) Clustering STP values to obtain the STP Bins, and those values were divided into various bins defining the equivalent interval as 1 kW/ton, as shown in Equation (2):

(2) $$\forall :{\mathrm{STP}}_{t}\in [n-0.5,n+0.5]\;;\;\mathrm{STP}\;\mathrm{Bin}\;\#=n\;;\;n\in [-20,20]$$

where STP Bin # = ID of the STP pool, and *n* = an integer

Obtaining STP distributions by computing the time percentage in each STP bin within a defined speed interval for unloaded, fully loaded, and overloaded trucks. The continuous speed data were divided into trajectories, each consisting of 60 consecutive seconds of operating data. The average speed was calculated for each trajectory using Equation (3). Then, the trajectories were put into trajectory pools with specific speeds based on their average speed. Meanwhile, the specific-speed trajectory pool was defined by the speed bin with an equal spacing of 2 km/h, as shown in Equation (4):

(3) $$\mathrm{average}\;\mathrm{speed}=3.6\;\cdot \;\sum_{i=1}^{60}v_{i}/60$$

(4) ∀:average speed ∈(n−2,n]; Speed Bin# = n

The mean and standard deviation were calculated separately for each pollutant in the STP Bin. The mean value plus and minus three times the standard deviation was used as the threshold value within each STP bin to ensure that the second-by-second emission rate data were within the corresponding threshold values. After removing abnormal data, the average emission value in each STP bin was taken as the pollutant’s emission rate. The average emission rate was calculated using Equation (5) as follows:

(5) $$ER_{ij}=\sum_{n_{ij}=1}^{N_{ij}}ER_{ijn}/N_{ij}$$

where *ER_ij_*= average emission rate of emission *i* in STP bin *j* (g/s), *ER_ijn_* = the *n*-th measured value of emission *i* in STP bin *j* (g/s), and *N_ij_* = the amount of data of emission *i* in STP bin *j*.

### 2.3. The Calculation Method of Emission Factor Based on STP Distribution and Emission Rate

To contrast and characterize differences in emissions of DSTTTs under varied loads further, emission factors for trucks with different load states were calculated. First, the STP distribution in each speed bin was used to describe driving behaviors based on field operation data. Then, emission rates for different load states in STP bins were calculated according to collected emission data. Finally, taking STP as the intermediate parameter, the STP distribution and emission rate were integrated to calculate emission factors for different loaded trucks at different speed intervals using Equation (6):

(6) $$EF_{j}(v_{k})=\sum_{i=-20}^{20}[ER_{ij}\times STPDistribution_{i}(v_{k})]\times 3.6/(v_{k}-1)$$

where *EF_j_(v_k_)* = the emission factor of *j* for trucks at the speed interval of *v_k_* (g/km), *ER_ij_* = the emission rate of *j* for trucks at the STP bin of *i* (g/s), *STPDistribution*(*v_k_*) = the percentage of STP Bin of *i* for trucks at the speed interval of *v_k_* (%), and *v_k_* = the speed bin (m/s).

## 3. Results and Discussion

### 3.1. STP Characteristics for DSTTTs under Different Load States

**Figure 4:**
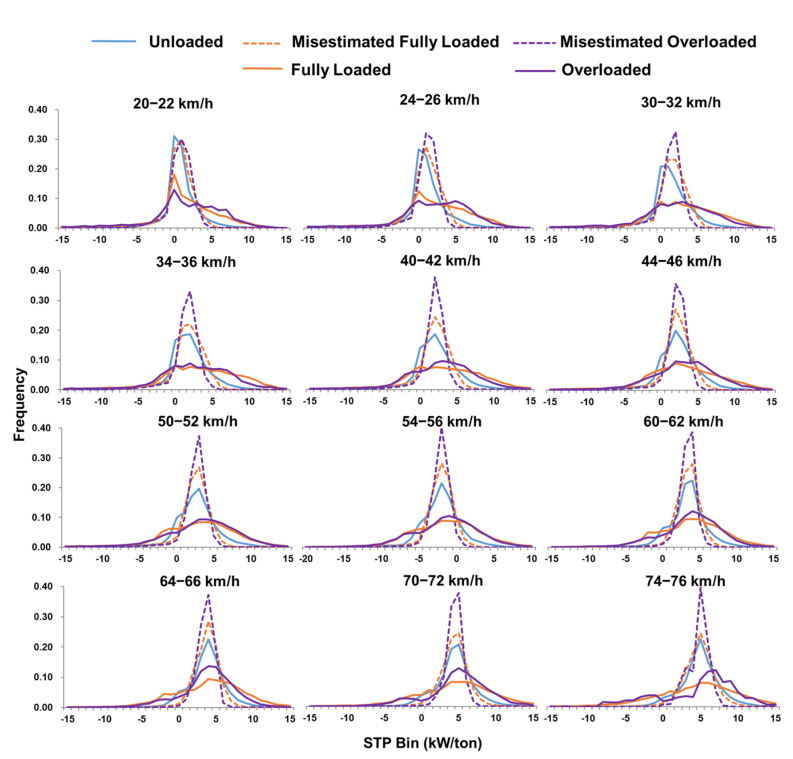
STP distribution for DSTTTs with unloaded, fully loaded and overloaded in different speed bins (unloaded, m = 14.5 tons; misestimated fully loaded, *m* = 14.5 tons; misestimated overloaded, *m* = 14.5 tons; fully loaded, *m* = 49.0 tons; overloaded, m = 61.5 tons).

The STP distribution is the percentage of time that the vehicle spends in each STP bin during operation, representing the power needed for a given operating mode. To analyze the STP distribution characteristics of DSTTTs under various loading states, 12 speed ranges were selected for analysis, which were 20 to 22 km/h, 24 to 26 km/h, 30 to 32 km/h, 34 to 36 km/h, 40 to 42 km/h, 44 to 46 km/h, 50 to 52 km/h, 54 to 56 km/h, 60 to 62 km/h, 64 to 66 km/h, 70 to 72 km/h, and 74 to 76 km/h, respectively, as shown in Figure 4. However, it was difficult to obtain real-time load information of trucks on the road network when evaluating the emissions of road network trucks, which led to mistaking fully loaded and overloaded trucks as empty. In most cases, the STP distribution of the truck under fully loaded and overloaded conditions was calculated according to the unloaded truck weight. This study also analyzed the STP distribution characteristics when the fully loaded or overloaded trucks were mistaken as the unloaded state, as shown in Figure 4.

**Figure 5:**
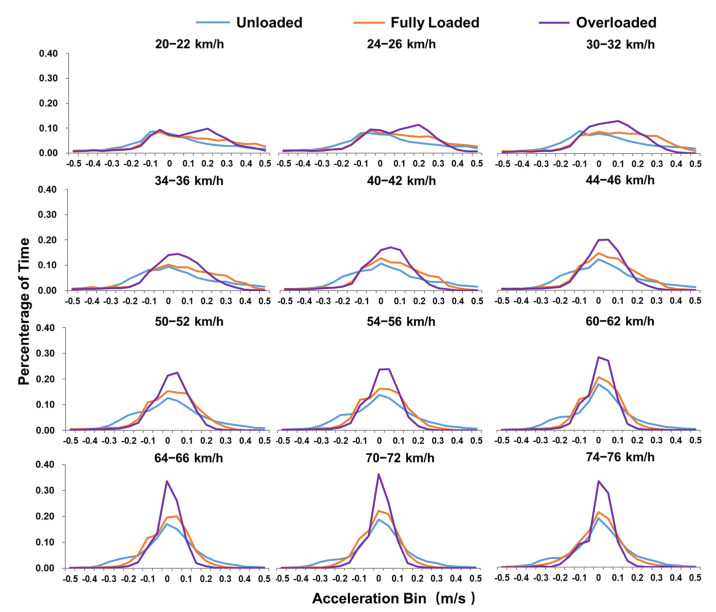
Acceleration distribution for DSTTTs with unloaded, fully loaded and overloaded in different speed bins.

STP distributions at speed intervals under different load statuses were also illustrated in Figure 4, and the characteristics could be summarized as follows:

(1) STP distributions within all the speed intervals were shaped like a Gaussian distribution, regardless of load state. Under the same load condition, the STP distribution was more cramped as the speed increased. The STP distributions of fully loaded and overloaded conditions obtained from the actual vehicle weight were quite different from those obtained from the unloaded vehicle weight.
(2) When the STP distribution was studied according to the actual vehicle weight, the STP distribution under the unloaded state was more concentrated than that under the fully loaded or overloaded state. The lower part of the Gaussian distribution for fully loaded and overloaded trucks was significantly wider than that for trucks with an unloaded state. The peak value of the STP distribution within the unloaded state was significantly higher than that of the fully loaded or overloaded state. In speed intervals of less than 32 km/h, the STP distribution percentages of full load and overload were greater than those of the load in the higher bins. Meanwhile, in the speed intervals greater than 34 km/h, the percentages of STP distributions for fully loaded and overloaded were greater than those for the unloaded condition in both the higher and lower bins. Besides, the STP distribution of the overloaded state was more cramped than that for the fully loaded state, especially when the speed was greater than 64 km/h.
(3) When the STP distribution was analyzed according to the unloaded truck weight under the conditions of fully loaded and overloaded, the STP distributions were more concentrated as the truck load increased in the same speed interval. This was mainly because as the load increases the acceleration and deceleration performance of the vehicle became worse, making the acceleration distribution more concentrated, as shown in Figure 5. In addition, the peak value of STP distribution shifted to the right as the average speed increased. At the speed intervals of less than 32 km/h, the bins with the highest percentage of STP distributions for the empty, full load, and overload conditions were different and the distributions of full load and overload were shifted more to the right. The highest percentage of STP distributions for empty, full load, and overload conditions were within the same bins in the speed intervals analyzed for speeds of greater than 34 km/h.

### 3.2. Emission Rate Analysis of Different Load States

**Figure 6:**
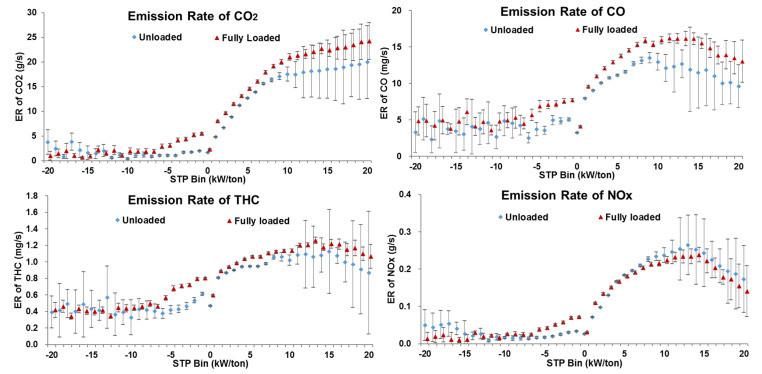
Emission rates of CO_2_, CO, THC and NO_x_ for DSTTTs with unloaded and fully loaded in STP bins. (with 95% confidence intervals).

The emission rate model is an essential tool that can be used in conjunction with vehicle activity data to assess the effect of traffic-control strategies on emissions. One significant factor that affects the accuracy of emissions assessments is the difference in vehicle emission rates under various vehicle load conditions. Therefore, this study collected emission data of CO_2_, CO, THC, and NO_x_ for 15 unloaded and fully loaded DSTTTs. Due to the limitations of collection conditions, the emissions data of overloaded vehicles could not be obtained. In this study, the emission differences between unloaded and fully loaded vehicles in each bin were compared, as shown in Figure 6. To quantify emission discrepancies between fully loaded and empty trucks, a parameter α representing the relative difference in emission rates between fully loaded and unloaded DSTTTs was defined in Equation (7), while the differences of α in emission rates between empty and fully loaded trucks are shown in Table 3:

(7) $$\alpha_{ij}=\frac{ER_{fullyloaded-ij}-ER_{unloaded-ij}}{ER_{fullyloaded-ij}}\times 100\%$$

where, *α_ij_* = the relative difference of *j* emission rates between fully loaded and unloaded DSTTTs in the STP Bin *i* (%), *ER_fullyloaded-ij_* = the *j* emission rate of DSTTTS under fully loaded status in the STP bin of *i* (g/s), and *ER_unloaded-ij_* = the *j* emission rate of the truck under unloaded status in the STP sin of *i* (g/s).

**Figure 7:**
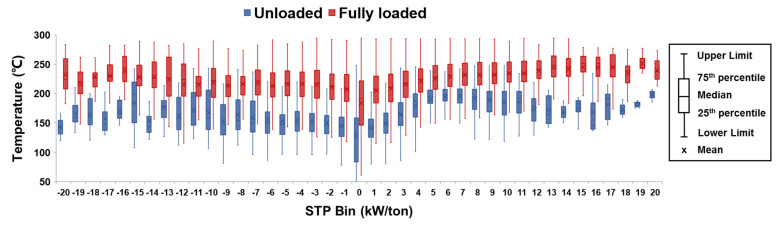
The difference in exhaust temperature between fully loaded and unloaded trucks. (IQR = 75th percentile − 25th percentile, Lower Limit = max (minimum in the sample, 25th percentile − 1.5IQR), Upper Limit = min (maximum in the sample, 75th percentile + 1.5IQR)).

According to Figure 6 and Table 3, the characteristics of the emission rate difference of trucks under unloaded and fully loaded conditions could be expounded as follows:

(1) When the STP bin was less 0 kW/ton, the emission rate differences of CO_2_, CO, THC, and NO_x_ between the fully loaded and unloaded trucks were consistent between pollutants. When the STP bin was −11 kW/ton or less, each pollutant’s emission rate was relatively low, and α shows positive and negative values. When the STP bin was between −10 kW/ton and −1 kW/ton, the emission rates of the DSTTTs in the fully loaded state in each bin were significantly higher than those in the unloaded state, especially when the STP bin was −5 kW/ton or greater. When the STP bin was between −5 kW/ton and −1 kW/ton, the average values of α for CO_2_, CO, THC, and NO_x_ were 209.9%, 67.7%, 52.6%, and 137.3%, respectively.
(2) When the STP bin was 0 kW/ton or greater, the emission rates of CO_2_, CO, THC, and NO_x_ showed diverse trends together with bins increases. With increases in the STP bin, the CO_2_ emission rates of trucks in fully loaded and unloaded states showed a rapid increase and then the growth rate slowed down. In each bin, the CO_2_ emission rate of fully loaded DSTTTS was higher than that of unloaded. The average values of α were 27.6% and 22.0% when the STP bin was between 0 kW/ton and 10 kW/ton and between 11 kW/ton and 20 kW/ton, respectively.
(3) When the STP bin was 0 kW/ton or greater, the CO emission rates of vehicles in fully loaded and unloaded states showed a rapid increase but then a decrease increasing STP bin. In each bin, the CO emission rate of fully loaded DSTTTS was higher than of unloaded vehicles. The average values of α were 21.0% and 32.2% when the STP bin was between 0 kW/ton and 10 kW/ton and between 11 kW/ton and 20 kW/ton, respectively.
(4) When the STP bin was 0 kW/ton or greater, the THC emission rates of fully loaded and unloaded vehicles showed the trend of increasing first and then decreasing slightly as the STP bin increased. In each bin, the THC emission rate of fully loaded DSTTTs was higher than that of unloaded DSTTTs. The average values of α were 12.2% and 15.1% when the STP bin was between 0 kW/ton and 10 kW/ton and between 11 kW/ton and 20 kW/ton, respectively.
(5) Finally, when was 0 kW/ton or greater, the NO_x_ emission rates of fully loaded and unloaded vehicles displayed the trend of increasing first and then decreasing sharply as the STP bin increased. When the STP bin was between 0 kW/ton and 3 kW/ton, the NO_x_ emission rate of fully loaded DSTTTs was higher than that of unloaded trucks and the average value of α was 31.3%. However, when the STP bin was 4 kW/ton or greater, the NO_x_ emission rate of a fully loaded DSTTTS was lower than that of an unloaded vehicle. The absolute value of α increased gradually with increasing STP bin, and the average value of α was −8.6%. To further analyze the reasons for this variation in NO_x_ emission rate, this study investigated the difference in vehicle exhaust temperature between unloaded and fully loaded conditions. For DSTTTs equipped with an SCR system, the exhaust temperature was an important factor affecting NO_x_ conversion efficiency. For instances where SCR uses vanadia as a catalyst, the NO_x_ conversion efficiency gradually increases with increasing temperature when the exhaust temperature is lower than 350 °C [25,26]. As shown in Figure 7, the exhaust temperature of DSTTTs in the fully loaded state was significantly higher than that of those in an unloaded state, which was why the NO_x_ emission rate of vehicles in the fully loaded state was lower than that in the unloaded state.

### 3.3. Characteristics of Emission Factors for DSTTTS under Different Loading Conditions

**Figure 8:**
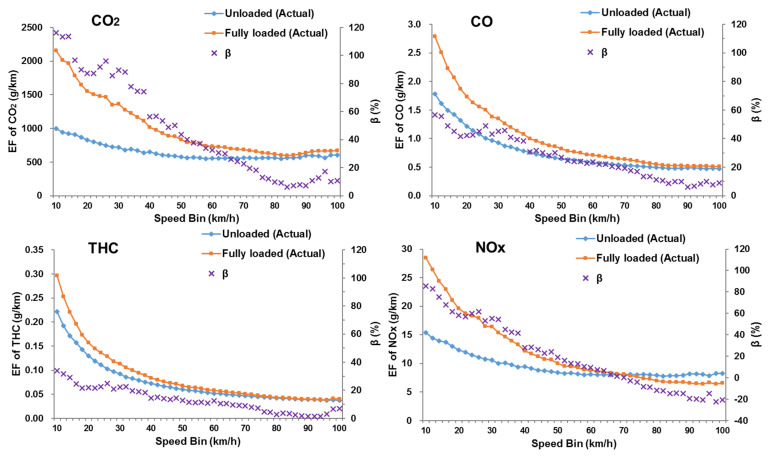
The emission factor differences of CO_2_, CO, THC and NO_x_ between fully loaded and unloaded status in each speed bin.

**Figure 9:**
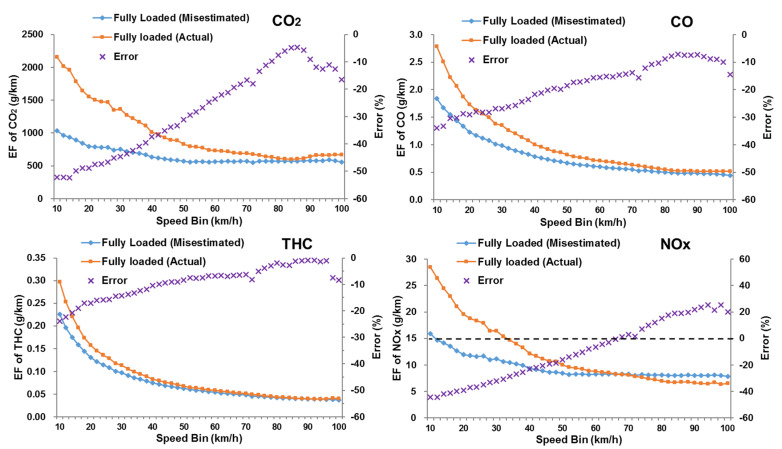
The emission factor differences of CO_2_, CO, THC and NO_x_ between fully loaded and misestimated-fully loaded status in each speed bin. (emission factor of misestimated-fully loaded states was obtained by calculating the STP distribution using the m of unloaded trucks and combining with the emission rate of unloaded trucks.).

Based on the calculation methods in the previous section, this study analyzed differences in the emission factors between unloaded and fully loaded trucks in each speed range, as shown in Figure 8. A variable β (%) represented the relative difference of emission factors between fully loaded and unloaded DSTTTs. It was difficult to obtain real-time load information of all trucks on the road network, resulting in the full-load state often being mistaken for the empty load status. Therefore, in this study, the misestimated fully loaded emission factor was obtained by calculating the fully loaded STP distribution using the unloaded weight and combining the unloaded trucks’ emission rate, as shown in Figure 9. The calculation method of the emission factor measurement error caused by the fully loaded state being mistaken for the no-load state was shown in Equation (8):

(8) $$Error_{j}(v_{k})=\frac{EF_{misestimated\;fullyloaded-j}(v_{k})-EF_{fullyloaded-j}(v_{k})}{EF_{fullyloaded-j}(v_{k})}\times 100\%$$

where, *Error_j_*(*v_k_*) = the relative difference of *j* emission factors between fully loaded and misestimated-fully loaded DSTTTs at the speed bin *v_k_* (%), *EF_fullyloaded-j_*(*v_k_*) = the emission factor of *j* for fully loaded trucks at the speed bin *v_k_* (g/km), and *EF_misestimated fullyloaded-j_*(*vk*) = the emission factor of *j* for misestimated-fully loaded trucks at the speed bin *v_k_* (g/km).

In Figure 8 and Figure 9, the emission factors of all pollutants decreased as the operating speed increased. When the speed bin was 40 km/h or less, the emission factor dropped sharply as the speed increased, while, when the speed was 40 km/h or greater, the downward trend of the emission factor slowed down. There were specific noticeable differences noted between unloaded and fully loaded trucks as follows:

(1) For CO_2_, CO, and THC, the emission factors of the fully-loaded DSTTTs in each speed bin were higher than those of the unloaded trucks. However, the NO_x_ emission factors of the fully-loaded trucks were higher than that of the unloaded trucks when the speed bin was 66 km/h or less, while the situation was opposite when the speed bin was 68 km/h or greater.
(2) The analysis results revealed that the relative difference β (%) in emissions between different load states was closely related to truck operating speed. The loading state had the most significant effect on the CO_2_ emission factors and the weakest impact on THC emission factors. When the operating speed was low (0–30 km/h), the load had the most significant impact on the emission factor, with β (%) values ranging from 27.8% to 96.2%.
(3) The impact of the load on emissions gradually lessened with increasing speed. Since the power required by the truck to overcome aerodynamic drag increased ternary to the operating speed, the effect of the load on the power was gradually weakened. When the operating speed was medium (30–60 km/h), the β (%) values of CO_2_, CO, THC, and NO_x_ were 53.7%, 30.8%, 14.8%, and 26.3%, respectively. When the operating speed was high (60–100 km/h), the NO_x_ emission factor of the fully-loaded vehicle was lower than that of the unloaded vehicle, and the β (%) values of CO_2_, CO, THC, and NO_x_ were 15.3%, 13.1%, 5.4%, and −9.5%, respectively.
(4) Mistaking the fully-loaded truck as being unloaded would seriously affect the result of emission measurement, as shown in Figure 9. In this context, at low speeds (0–30 km/h), the emissions factors of CO_2_, CO, THC, and NO_x_ were underestimated by 48.1%, 30.1%, 19.9%, and 38.2%, respectively. The average emission factor errors of CO_2_, CO and THC were −29.6%, −19.5% and −10.5%, respectively. When the speed bin was 66 km/h or less or the speed bin was 68 km/h or greater, the average NO_x_ emission factor errors were −26.1% and 15.4%, respectively.

## 4. Conclusions

STP distribution and emission rates are crucial factors affecting the emission of DSTTTs under different load states. An emission model of DSTTTs under different load states was established according to the field operating data collected by OBD and the emissions data obtained by PEMS. This study refined the STP computing method according to the load, making it practicable to analyze emissions for on-road trucks with various load states in the same traffic scenarios. Differences in STP distributions, emission rates, and emission factors for DSTTTs at various loads were analyzed. The most significant findings from this study can be summarized as follows:

(1) Mistaking the fully loaded vehicle as an unloaded one would significantly misestimated DSTTTs emissions. The emission factors of CO_2_, CO, and THC were underestimated in all speed bins, while the emission factor of NO_x_ was overestimated at speeds greater than 68 km/h. The largest errors in emission measurements were found in low speeds (0–30 km/h), where average emission factors for CO_2_, CO, THC, and NO_x_ were underestimated by 48.1%, 30.1%, 19.9%, and 38.2%, respectively.
(2) There were significant differences in STP distributions for various loaded DSTTTs. The STP distribution of unloaded DSTTTs was more concentrated than that of the fully loaded and overloaded conditions. The peak value of STP distribution for the unloaded state was significantly higher than those for the fully loaded and overloaded states. The bottom of the Gaussian distribution for fully loaded and overloaded trucks was obviously wider than that for trucks with no loads.
(3) When the STP bin was −10 kW/ton or greater, the emission rates of CO_2_, CO, and THC for DSTTTs under fully loaded state were higher than those for the vehicles under an unloaded state within each STP bin. However, the NO_x_ emission rate under fully loaded condition was not necessarily higher than that under the unloaded condition due to the influence of exhaust temperature. The NO_x_ emission rate for DSTTTs in a fully loaded state was higher than that for trucks in an unloaded state when the STP bin was between −10 kW/ton and 3 kW/ton, while the situation was opposite when the STP bin was 4 kW/ton or greater.
(4) The emission factors of DSTTTs evolving from unloaded to fully loaded were significantly different. The emissions factors of CO_2_, CO, THC, and NO_x_ for fully loaded trucks increased the most at low speeds (0 to 30 km/h). The average growth rates of CO_2_, CO, THC, and NO_x_ were 96.2%, 47.9%, 27.8%, and 65.2%, respectively. As the operating speed increased, the effect of load on emissions gradually weakened. On average, the growth rates of emission factors for fully loaded trucks versus unloaded trucks were 15.3% for CO_2_, 13.1% for CO, and 5.4% for THC at low speeds (60 to 100 km/h), while, conversely, the emissions factors of NO_x_ for fully loaded trucks decreased by 9.5% due to the influence of exhaust temperature.

The emissions model proposed in this study is able to evaluate the differences in emission associated with various loading conditions and provided a potential method to adjust vehicle emissions according to load weight and traffic conditions. However, this study failed to obtain emissions data of DSTTTs in an overloaded state, and the effect of road type on DSTTTs’ emissions has not been discussed. It is necessary to conduct a more in-depth study on more fine-grained load states and road types in the future.

## Figures and Tables

**Table 1:** Basic information about DSTTTs for speed data collection.

Vehicle ID	Model	Fuel Type	Power (kW)	Engine Size (L)	Curb Weight (Tons)	Gross Vehicle Weight (Tons)	Field Load (Tons)
1	SX4250MB4	diesel	276	9.5	8.6	40.0	14.5/49.0/61.5
2	SX4250MB4	diesel	276	11.6	8.6	40.0	14.5/49.0/61.5
3	BJ4252SNFKB-XA	diesel	257	10.5	7.3	38.5	14.5/49.0/61.5
4	BJ4253SNFKB-XJ	diesel	279	11.8	7.3	38.5	14.5/49.0/61.5
5	LZ4240H7CA	diesel	316	11.6	7.3	38.5	14.5/49.0/61.5
6	C4250P66K2471A1E5	diesel	342	11.0	8.8	40.0	14.5/49.0/61.5
7	CGC4252D4XBA	diesel	316	11.6	8.0	37.8	14.5/49.0/61.5
8	CGC4250D5EDDE	diesel	316	11.6	8.8	40.0	14.5/49.0/61.5
9	SX4250MC4	diesel	294	9.5	8.6	40.0	14.5/49.0
10	GX4256GR129	diesel	247	9.7	7.2	38.6	14.5/49.0/61.5

**Table 2:** The parameters of DSTTTs for emission data collection.

ID	VIN	Vehicle Model	Model Year	Fuel Type	Emission Standard	Power (kW)	Engine Size (L)	Test Date (Day/Month/Year)	Field Load (tons)
1	LGGA40X36GL***447	LZ4230G2CA	2016	diesel	National 4	226	9.7	23/08/2018	14.5/49.0
2	LGGA4DX31GL***240	LZ4230G2CA	2016	diesel	National 4	226	9.7	24/08/2018	14.5/49.0
3	LGGA4DX32GL***621	LZ4230G2CA	2016	diesel	National 4	226	9.7	25/08/2018	14.5
4	LGGA4DX32GL***451	LZ4230G2CA	2016	diesel	National 4	226	9.7	27/08/2018	14.5/49.0
5	LGGA4DX33GL***132	LZ4230G2CA	2016	diesel	National 4	226	9.7	28/08/2018	14.5
6	LGGA4DX34GL***460	LZ4230G2CA	2016	diesel	National 4	226	9.7	29/08/2018	14.5
7	LGGA4DX34GL***670	LZ4230G2CA	2016	diesel	National 4	226	9.7	30/08/2018	14.5/49.0
8	LGGA4DX34GL***298	LZ4230G2CA	2016	diesel	National 4	226	9.7	31/08/2018	14.5
9	LGGA4DX34GL***387	LZ4230G2CA	2016	diesel	National 4	226	9.7	03/09/2018	14.5
10	LGGA4DX34GL***435	LZ4230G2CA	2016	diesel	National 4	226	9.7	04/09/2018	14.5
11	LGGA4DX34GL***525	LZ4230G2CA	2016	diesel	National 4	226	9.7	04/09/2018	14.5
12	LGGA4DX36GL***383	LZ4230G2CA	2016	diesel	National 4	226	9.7	04/09/2018	14.5/49.0
13	LGGA4DX39GL***003	LZ4230G2CA	2016	diesel	National 4	226	9.7	07/09/2018	14.5/49.0
14	LGGA4DX3XGL***460	LZ4230G2CA	2016	diesel	National 4	226	9.7	10/09/2018	14.5
15	LGGA4DX3XGL***543	LZ4230G2CA	2016	diesel	National 4	226	9.7	10/09/2018	14.5

**Table 3:** The difference in emission rate under different loading conditions.

STP Bin	α (%)			
	CO_2_	CO	HC	NO_x_
−20	−74.0	46.1	6.4	−75.0
−19	−41.8	−4.6	12.0	−56.7
−18	135.5	80.0	−30.4	−54.1
−17	−74.5	2.3	8.6	−79.4
−16	−70.3	0.5	−16.0	−76.8
−15	−41.8	37.7	−6.7	−56.9
−14	59.4	99.4	0.6	41.3
−13	−26.1	0.8	−39.6	−27.8
−12	262.8	24.9	22.2	151.6
−11	−1.7	−21.5	11.9	−12.5
−10	391.3	79.2	35.4	65.6
−9	75.3	1.5	8.1	76.0
−8	55.3	16.8	19.2	45.0
−7	116.3	5.1	13.8	79.0
−6	181.3	126.9	50.6	140.1
−5	186.2	85.4	62.0	150.7
−4	312.1	98.	65.5	158.1
−3	166.9	45.6	55.2	130.4
−2	205.3	56.5	49.1	132.7
−1	178.9	52.6	31.4	114.5
0	42.3	27.4	27.8	19.6
1	68.1	20.9	10.7	53.0
2	46.0	21.9	8.6	36.8
3	30.1	20.1	10.3	15.8
4	17.2	20.3	10.5	−0.4
5	15.3	23.8	13.2	−2.0
6	15.6	25.1	13.1	−3.1
7	15.1	20.8	13.6	−3.4
8	14.3	20.8	6.7	−6.7
9	15.5	13.7	7.6	−8.6
10	19.6	22.8	11.9	−5.9
11	17.9	33.5	10.7	−6.1
12	26.4	31.2	10.6	−8.7
13	19.4	27.3	18.8	−11.9
14	28.8	35.3	9.2	−6.1
15	18.1	34.7	8.5	−9.4
16	26.0	25.2	13.3	−10.3
17	18.2	26.0	15.5	−14.9
18	21.9	39.1	20.7	−11.8
19	30.0	33.6	21.1	−18.0
20	26.7	35.7	23.1	−19.1

## Data Availability

Data sharing not applicable.
